# Prevalence of *Corynosoma caspicum* infection in *Gasterosteus aculeatus* fish in Caspian Sea, Northern Iran

**DOI:** 10.14202/vetworld.2017.1139-1142

**Published:** 2017-09-27

**Authors:** Bahman Rahimi-Esboei, Mahdi Najm, Morad Shaker, Mahdi Fakhar, Iraj Mobedi

**Affiliations:** 1 Department of Parasitology and Mycology, School of Public Health and Health Research Institute, Tehran University of Medical Sciences, Tehran, Iran; 2 Department of Parasitology, Student Research Committee, School of Medicine, Iran University of Medical Sciences, Tehran, Iran; 3 Department of Fisheries, Islamic Azad University, Babol Branch, Babol, Iran; 4 Department of Parasitology, Molecular and Cell Biology Research Center, School of Medicine, Mazandaran University of Medical Sciences, Sari, Iran

**Keywords:** Acanthocephalosis, Caspian Sea, *Corynosoma caspicum*, *Gasterosteus aculeatus*

## Abstract

**Aim:**

There is little information about the prevalence of *Corynosoma caspicum* in fish particularly *Gasterosteus aculeatus* in Iran and the world. The aim of the present study was to find out the prevalence of acanthocephalan infection in Babolsar district, southern coastal of Caspian Sea, Northern Iran.

**Materials and Methods:**

Between September 2012 and August 2014, a total of 360 *G. aculeatus* fishes were randomly collected by drift nets from coastal regions in Babolsar and then examined the intestine and body cavity for worm infections.

**Results:**

A total of 360 *G. aculeatus* fishes, 109 (30.3%) were found infected with at least one *Corynosoma capsicum*, and there was no significant association between genders and the prevalence infection of acanthocephalan. Moreover, there was a significant difference in infected rate between summer (79%, 86/109) and spring (21%, 23/109) (p<0.05).

**Conclusion:**

The high occurrence of *Corynosoma* infection in *G. aculeatus* indicates the enzootic constancy status of the infection in the southern coastal of Caspian Sea, Northern Iran.

## Introduction

Acanthocephala is a phylum of parasitic worms known as acanthocephala, and the phylum is included in four classes - Palaeacanthocephala, Archiacanthocephala, Polyacanthocephala, and Eoacanthocephala [1]. Thorny-headed worm or spiny-headed worms have variable length from 6 mm to 30 cm. Proboscis is retractable into the body; body covered with tegument and spines and absorbs the nutrients. Its life cycle routinely requires only one intermediate host, such as cyclops or brine shrimp [2]. Acanthocephalans can infect a number of hosts including fishes, amphibians, birds, and mammals as final hosts. Some species of acanthocephalans (known as the perforating acanthocephalan) can pierce their proboscis in the gut wall of the host and go through to the peritoneal cavity [3]. To date, about 1150 species of acanthocephalans have been described by Cleave [4].

*Gasterosteus aculeatus* is a kind of three spine sticklebacks; these fishes have three dorsal spines, two pelvic spines, and one anal spine [5]. It is common in slow-moving backwaters of rivers, lakes, ponds, sheltered bays, and harbors with a worldwide distribution in coastal marine and fresh water of the northern hemisphere [6]. *Corynosoma caspicum* belongs to the class of Palaeacanthocephala [7], order of Polymorphida [8], family of Polymorphidae [7], and genus and species of *C. caspicum* [8]. Recently, a comprehensive list relating to the acanthocephalan fauna of Iran has been reported. In total, 30 known species of acanthocephala from 21 genera, 12 families, and 7 orders were reported from 80 species of various vertebrates in Iran [9]. Species identification of acanthocephalans is mostly based on morphological features, including the size and number of hooks on the proboscis and the number of spines on the body surface [3,4].

Although *Corynosoma strumosum* and *Bolbosoma caenoforme* have been previously reported in the *G. aculeatus* fish from north of Iran [10,11], *C. caspicum* has been reported from different fish species except *G. aculeatus* fish. In fact, little information is available regarding prevalence and density of *C. caspicum* among fishes in Iran and around the world principally Caspian Sea littoral countries. Thus, the aim of the present study was to determine the prevalence and intensity of acanthocephalan infections, in *G. aculeatus* fishes in the Southern part of the Caspian Sea.

## Materials and Methods

### Ethical approval

During all stages of our research, all applicable international, national, and/or institutional guidelines for the care and use of animals were followed. The fishes were caught according to the ethical approval of the Caspian Sea Ecology Research Center.

### Study area

Between 2012 and 2014, the present study was conducted in southern coastal of Caspian Sea, Mazandaran Province (53°6′5E, 36°23′3N). This province has a moist and temperate weather with an average annual rainfall of about 1000 mm [12].

### Fish collection and biometric measuring

In this study, 360 *G. aculeatus* fish samples were collected by fishermen, using drift nets with 5 mm mesh size, during September 2012-August 2014 in coastal Caspian Sea in Babolsar district, Northern Iran. After being caught, the fishes were frozen at −20°C and transported to the Caspian Sea Ecology Research Center Laboratory, and biometric features such as total length, standard length, and sex for each fish were measured [2].

### Fish examination for the acanthocephala

The fish samples were subsequently dissected, and the lumen of the intestine of them was examined for acanthocephalan parasites and then morphological features using a stereomicroscope with several magnifications. At first, collected acanthocephalans were washed in normal saline solution, irrigated in water to emerge the proboscis overnight. Later, the worms were fixed in alcohol/formalin/acetic acid fixative, preserved in 70% ethanol until processed for identification. Next, they were cleared with lacto phenol and then stained with azocarmine stain. Morphological and morphometric characteristics for every specimen were drawn by camera lucida, at 400× magnification and characterization were confirmed based on key references [13,14]; in addition, the parasite specimens were sent to Iranian Parasitology National Museum (IPNM) at Veterinary School of Tehran University for species confirmation. Our study was a descriptive cross-sectional study; hence, a negative control was not included.

## Results

A total of 360 *G. aculeatus* caught, 109 (30.3%) were infected for acanthocephalan throughout September 2012-August 2014. In total, 67% (73/109) and 33% (36/109) of the infected fishes were female and male, respectively. No significant difference between gender and the prevalence of acanthocephalan was observed (p>0.05). In general, 192 fishes were caught in spring, and 168 ones were caught in summer, and there was a significant difference in infected rate between summer (79%, 86/109) and spring (21%, 23/109) (p<0.05).

**Figure-1:**
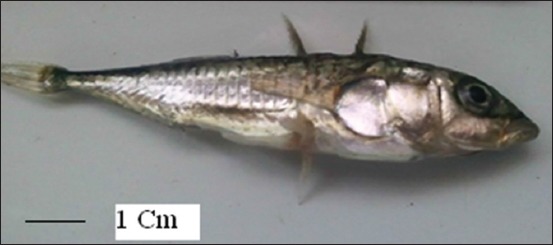
A male *Gasterosteus aculeatus* fish infected with *Corynosoma caspicus* from Caspian Sea; Note the dorsal spines on the body (scale bar: 1 cm).

Moreover, fish biometrical features showed the length mean±standard deviation (SD) was the 6±0.38 cm (Figure-1). No significant relationship was observed between fish size and the infection rate of acanthocephalan (p>0.05). The adult worms were found to be in the lumen of the intestine, and the majority of them attached to the intestinal mucosa though some in the peritoneal cavity. In addition, 24 out of 109 (22%) had numerous infections with more than two worm.

**Figure-2:**
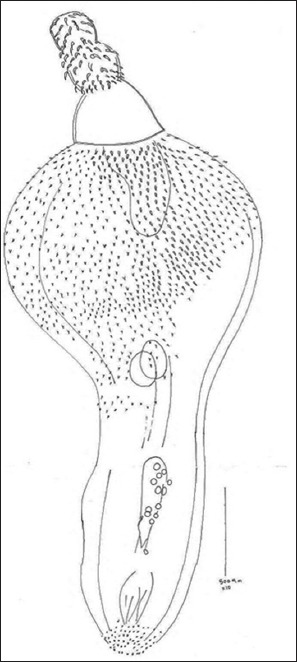
An adult male of *Corynosoma capsicum* showing anterior proboscis with longitudinal rows of hooks and the posterior proboscis with spines (scale bar: 500 µm).

Morphological features of the isolated adult acanthocephalan were as follow: The length mean±SD of adults was 4.3±2.7. The body comprises an anterior proboscis, a neck (presoma), and a trunk (metasoma) (Figure-2). There were 16-18 longitudinal rows of hooks and 10-11 hooks per row on the proboscis. The average size of hooks on proboscis was 5.96 µm and hooks on trunk were 2.73 µm. The end of posterior cone of the worms’ body was covered with five to six (mostly six) rows of spines. Based on these morphological details and the IPNM report [13,14], all specimens were identified as *C. caspicum*.

## Discussion

Lake fish parasites have received little notice in Iran; however, few studies have been performed in the recent years. In our study, for the first time, we found the high infection rate (30.3%) of *C. caspicum* in exotic *G. aculeatus* fish in coastal Caspian Sea, Northern Iran. The acanthocephalans parasites frequently cause intestinal perforation throughout heavy infected condition.

In this study, there was no significant difference between fish size and the prevalence of acanthocephalan. However, Amin *et al*. (2013) reported the prevalence of infection decreased with increasing fish size in case of *Acanthogyrus (Acanthosentis) barmeshoori* collected from the Persian tooth-carp, in Southern Iran. However, it seems that the relationship between prevalence of infection rate and size of fishes depends on species of fish and also acanthocephalans genera, although it remains controversial [15,16]. In general, there are no data in this view.

Furthermore, in our study, there is a significant difference between infection rate of *C*. *caspicum* and season. Seasonally, the prevalence of infection increased to 79% during summer season and decreased to 21% during spring; which our finding is in concurrence with the findings of comparable investigations [17]. However, little information is available on this subject in literature.

Although acanthocephalan infections are common in various regions of the world particularly marine sea; however, the prevalence rate of *C*. *caspicum* in fish is mainly unknown in Iran. Several studies have been conducted on fish parasites in the Caspian Sea [18-22]. In Iran, also, several researchers have been investigated the parasitic infections in fishes in Southern coastal of Caspian Sea [10,23-29], but there is little information concerning *Corynosoma* spp. in various species of fish.

In Iran, the majority of reports concerning acanthocephalan infection, in particular, *Corynosoma* spp. belongs to edible fishes such as *Clupeonella cultriventris* and *Acipenser stellatus* as sea food throughout food chain [28,30]. However, there is little investigation on the prevalence of parasitic infections in the *G. aculeatus*, as non-suitable for eating fishes, in Iran and the world.

The results of the present study showed that acanthocephalan infections are notably more common (30.3%) in Caspian Sea in comparison with Persian Gulf. *C. strumosum* has already been reported from Acipenseridae, Neogobiidae, and Clupeidae in various areas of Iran [10,28,30,31].

## Conclusions

The high prevalence and intensity of *Corynosoma* infection in *G. aculeatus* demonstrate the enzootic situation of marine fish acathocephalosis in the southern coastal of Caspian Sea, Northern Iran; therefore, it needs a basic push to decrease the rate of infection conceivably by natural control in the future such as control of other marine pests. However, each acanthocephalan species can possibly have a capable effect on its ecosystem by ideals of its impacts on its host species at each level. As a whole, our preliminary study recommended further and broad explorations on the brine shrimp and or cyclops, as intermediate host, and also other fish species, waterfowls and sea seal, as final host, in several regions of coastal Caspian Sea. Moreover, further investigation on the morphology of *C. caspicum* using scanning electron microscopy is recommended in future.

## Authors’ Contributions

MF designed all steps of the study. BRE, MS, and MN collected samples of fish and examined throughout the study. IM illustrated Figure-2 and characterized the morphology of *Corynosoma*. MS characterized the *G. aculeatus* fish. MN took a photo from the *G. aculeatus* fish (Figure-1). MF and BRE prepared the tables of results and discussed it. BRE wrote the manuscript draft, and all authors have read, revised, and approved the final draft.
